# Aspartames Alter Pharmacokinetics Parameters of Erlotinib and Gefitinib and Elevate Liver Enzymes in Wistar Rats

**DOI:** 10.3390/ph15111400

**Published:** 2022-11-14

**Authors:** Hajer AlRasheed, Aliyah Almomen, Haya I. Aljohar, Maria Arafah, Rana Y. Almotawa, Manal A. Alossaimi, Nourah Z. Alzoman

**Affiliations:** 1 Department of Pharmaceutical Chemistry, College of Pharmacy, King Saud University, P.O. Box 22452, Riyadh 11495, Saudi Arabia; 2 Department of Pathology, College of Medicine, King Saud University, P.O. Box 2925, Riyadh 11421, Saudi Arabia; 3 Department of Pharmaceutical Chemistry, College of Pharmacy, Prince Sattam bin Abdelaziz University, P.O. Box 173, Alkharj 11942, Saudi Arabia

**Keywords:** aspartame, tyrosine kinase inhibitors, erlotinib, gefitinib, pharmacokinetics, liver enzyme, rat

## Abstract

**Background:** Erlotinib (ERL) and gefitinib (GEF) are extensively metabolized by CYP450 enzymes. Aspartame (ASP), an artificial sweetener, induces CYP2E1 and CYP3A2 enzymes in the brain and could increase liver enzymes. In this work, the influence of ASP on the pharmacokinetics (PK) of ERL and GEF in Wistar rats was evaluated. **Methods:** The PKs of ERL and GEF were evaluated after receiving 175 mg/kg or 1000 mg/kg of ASP for four weeks using UPLC-MS/MS. Levels of liver enzymes after four weeks of ASP consumption were also evaluated. **Results:** ASP 175 mg/kg was able to significantly alter levels of C_max_ (36% increase for ERL, 38% decrease for GEF), AUC_0–72_ (205% increase for ERL, 41% increase for GEF), and AUC_0–∞_ (112% increase for ERL, 14% increase for GEF). Moreover, ASP 175 mg/kg decreased the apparent oral clearance ERL and GEF by 58% and 13%, respectively. ASP 1000 mg/kg increased C_max_ of ERL by 159% and decreased GEF’s C_max_ by and 73%. Both AUC_0–72_ and AUC_0–∞_ were increased by ASP 1000 for ERL and decreased for GEF. CL/F decreased by 64% for ERL and increased by 38.8% for GEF. Moreover, data indicated that ASP significantly increased levels of liver enzymes within two weeks of administration. **Conclusions:** Although ASP 175 and 1000 mg/kg alter ERL and GEF PKs parameters, ASP 1000 mg/kg has the highest impact on most parameters. ASP 1000 mg/kg also can significantly increase activities of liver enzymes indicating the possibility of inducing liver injury. Therefore, it might be of clinical importance to avoid the administration of aspartame containing products while on ERL or GEF therapy.

## 1. Introduction

Lung cancer is of the most commonly diagnosed cancers worldwide and is the leading cause of mortality from cancer [1,2]. The most common lung cancer is the non-small cell lung carcinoma (NSCLC) which account for 84% of diagnosed cases [1]. At the present time, the mutation in the epidermal growth factor receptors (EGFR) represents the most well-established target in NSCLC [3]. Such evidence leads to the development of tyrosine kinase inhibitors (TKIs) targeting the EGFR’s intracellular tyrosine kinase domain, which showed a significant effect on patients with advanced-stage NSCLC harboring EGFR mutation [4]. Moreover, it has been demonstrated that TKIs, such as Gefitinib (GEF) and Erlotinib (ERL), have a notable benefit as a treatment for EGFR mutation-positive tumors compared to the traditional combined platinum-based chemotherapy, making the TKIs a first-line treatment in EGFR-mutated NSCLC [4,5]. Food and other beverages in the food industry are known to contain additives for flavoring and reducing caloric intake which can alter the pharmacokinetic (PK) parameter and increase exposure to ERL and GEF [6]. Aspartame, an N-L-alpha-aspartyl-L-phenylalanine, 1-methyl ester; alpha-ASP, is a prominent artificial sweetener which is used commonly instead of sugar in several products, including soft drinks, multivitamins chewable tablets, and cereals [6]. Aspartame exists in two forms: aspartame alpha and beta form [7]. Aspartame is a synthetic dipeptide made of aspartic acid and phenylalanine methyl ester and elicits its sweetness by binding to the G-protein-coupled sweet heterodimer receptors, more specifically the T1R2 extracellular venus-flytrap (VFT) domain [8].

Once aspartame is ingested, it is metabolized to aspartic acid and phenylalanine, and methanol by stomach enzymes (esterase and peptidase) in both the gastrointestinal lumen and the intestinal mucosal cells, and only the digested metabolites reach the systemic circulation [6,7]. Previous study indicated that ASP could increase liver enzymes such as alanine aminotransferase (ALT), and, in turn, raises the chances of drug interactions. It is possible that phenylalanine and aspartic acid induce such actions through binding to blood protein transporters and displacing drugs, interfering with drug actions, modifying drug targets on the cell membrane, or increasing metabolic abnormalities in geriatrics resulting in altered drug reactions [6].

Our previous study evaluating the effect of flavored water on the PKs of ERL and GEF indicated that FW could significantly alter the PKs of both drugs when they are co-administered [9]. Since Aspartame is one of the components of FW and to our knowledge no previous study has evaluated the effect of ASP levels on PKs of TKIs and hepatic CYP 450; in this study we place more focus on the influence of ASP on the PKs of FER and GEF in Wistar rats.

## 2. Results

### 2.1. The Effect of ASP on ERL’s PKs

**Figure 1:**
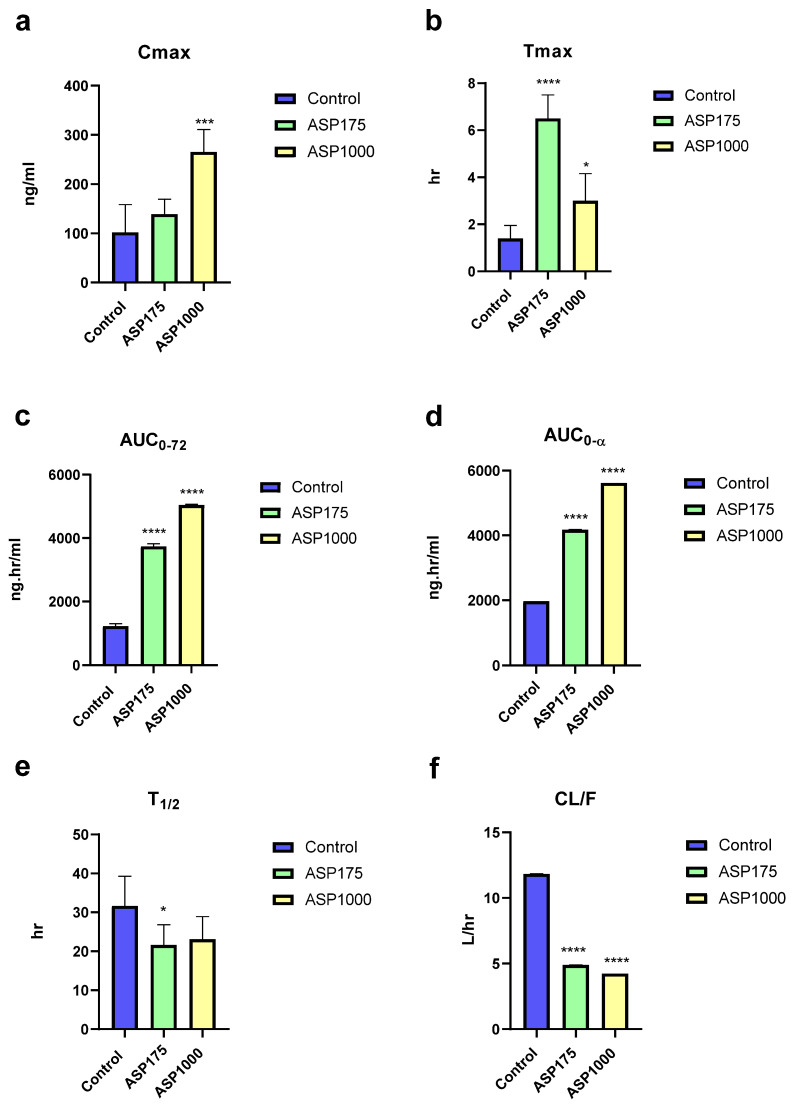
(**a**–**f**) Alteration in the main PK parameters (**a**) Cmax, (**b**) Tmax, (**_C_**) AUC_0-72_, (**d**) AUC_0-α_, (**e**) T_1/2_, (**f**) CL/F of ERL after four weeks administration of 1000 and 175 mg/kg ASP in rats compared to control (*n* = 5). *p*-values of 0.05 were considered statistically significance, where * *p* < 0.05, *** *p* < 0.001, and **** *p* < 0.0001.

After four weeks of ASP consumption, the result shows a significant increase in ERL’s C_max_ with 36% with ASP175 mg/kg and 159.7% with ASP1000 mg/kg (Figure 1a). Moreover, there was a 364% increase on T_max_ with ASP175 mg/kg and 114% increase with ASP 1000 mg/kg, (Figure 1b). On the other hand, ASP (175 and 1000 mg/kg) was able to significantly increase ERL AUC_0–72_ by 205% and 310% and AUC_0–∞_ by 112% and 185%, respectively (Figure 1c,d). Furthermore, an important change in the time required drug concentration to decrease by half for (T_1/2_) was found with almost 31% and 27% decreases with ASP 175 and 1000 mg/kg, respectively (Figure 1e). Regarding the apparent oral clearance, ASP 175 and 1000 mg/kg caused significant decreases of 58% and 64%, respectively (Figure 1f).

### 2.2. The Effect of ASP GEF’s PKs

**Figure 2:**
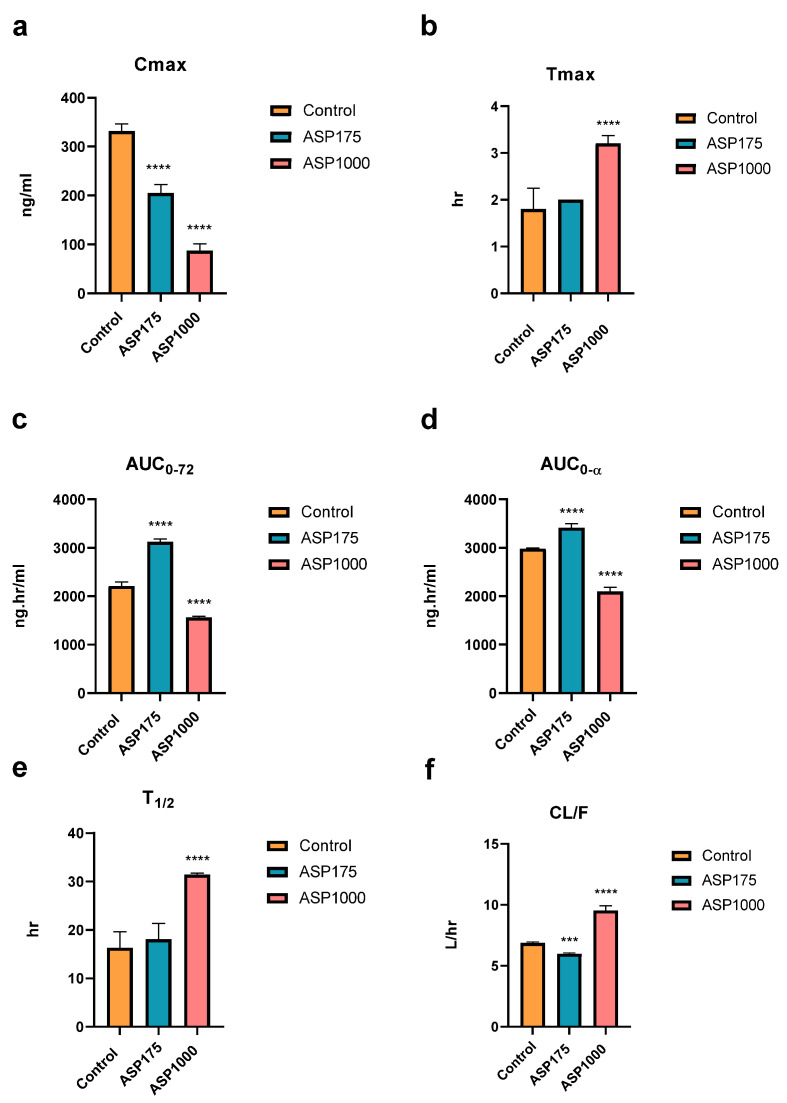
(**a**–**f**) Alteration in the main PK parameters (**a**) Cmax, (**b**) Tmax, (**_C_**) AUC_0-72_, (**d**)AUC_0-α_, (**e**) T_1/2_, (**f**) CL/F of ERL of GEF after four weeks administration of 1000 and 175 mg/kg ASP in rats compared to control (*n* = 5). *p*-values of 0.05 were considered statistically significance, where *** *p* < 0.001, and **** *p* < 0.0001.

Results indicate that after four weeks of ASP consumption followed by 20 mg/kg of GEF, a significant change in C_max_ relative to control was found. There was a 38% decrease in Cmax with ASP 175 mg/kg and a 73% decrease with ASP 1000 mg/kg (Figure 2a). Moreover, there was an almost 11% increase in T_max_ found with ASP 175 mg/kg and 77% with ASP 1000 mg/kg (Figure 2b). AUC_0–72_ was significantly increased by 41% by ASP 175 mg/kg and decreased by 29% by ASP 1000 mg/kg (Figure 2c). The same trend was found with AUC_0–∞_ where there was a 14.5% increase with ASP 175 mg/kg and 29.5% decrease with ASP 1000 (Figure 2d). ASP 175 and 1000 mg/kg caused an increase in T_1/2_ by 11% and 92%, respectively (Figure 2e). Regarding apparent clearance (CL/F), ASP 175 and 1000 mg/kg caused a significant decrease of 13%, and 38% increase, respectively (Figure 2f).

### 2.3. Liver Enzyme

**Figure 3:**
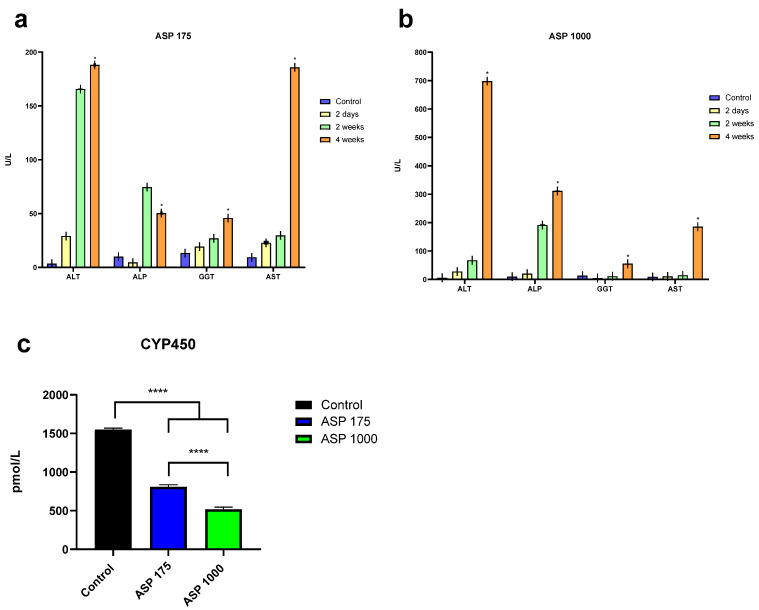
Liver enzyme concentration after four weeks of (**a**) ASP 175 mg/kg/day and (**b**) ASP 1000 mg/kg/day administrated rats, and (**c**) the effect of 4 weeks of ASP 175 and 1000 mg/kg/day on CYP 450 enzyme. Data are shown as mean ± SD (*n* = 5). *p*-values of 0.05 were considered statistically significance, where * *p* < 0.05 and **** *p* < 0.0001.

After four weeks of ASP consumption, the results show a significant increase in liver enzymes (ALT, ALP, GGT, and AST) with both doses ASP 175 and 1000 mg/kg (Figure 3a,b). Furthermore, a significant decrease in CYP 450 was found with both doses of ASP (Figure 3c).

### 2.4. Histopathology

Microscopic examination of liver tissues taken from rats receiving ERL only shows an intact lobular architecture with normal-looking hepatocytes radiating in one to two-cell-thick plates and scattered nuclei showing degenerative changes. A single rat in control showed foci of spotty necrosis. There was no evidence of massive or submassive necrosis. There were no signs of cholestasis, steatosis, fibrosis, bile duct injury, or vascular injury.

Sections from liver tissues taken from rats receiving ERL and ASP 175 mg/kg showed areas of sinusoidal congestion, degenerative nuclear changes and in a single rat and spotty necrosis with non-necrotizing granulomas. In addition to these changes, rats receiving ERL and ASP 1000 mg/kg showed a moderate degree of cytoplasmic vacuolization.

Microscopic examination of liver tissues taken from rats receiving GEF only shows an intact lobular architecture with normal-looking hepatocytes radiating in one to two-cell thick plates. Isolated apoptotic hepatocytes (acidophilic bodies) were present. Mild lobular and portal lymphocytic inflammation was noted in some rats. A single rat showed a vague non-necrotizing granuloma and focal spotty necrosis. There was no evidence of massive or submassive necrosis. There were no signs of cholestasis, steatosis, fibrosis, bile duct injury, or vascular injury.

**Figure 4:**
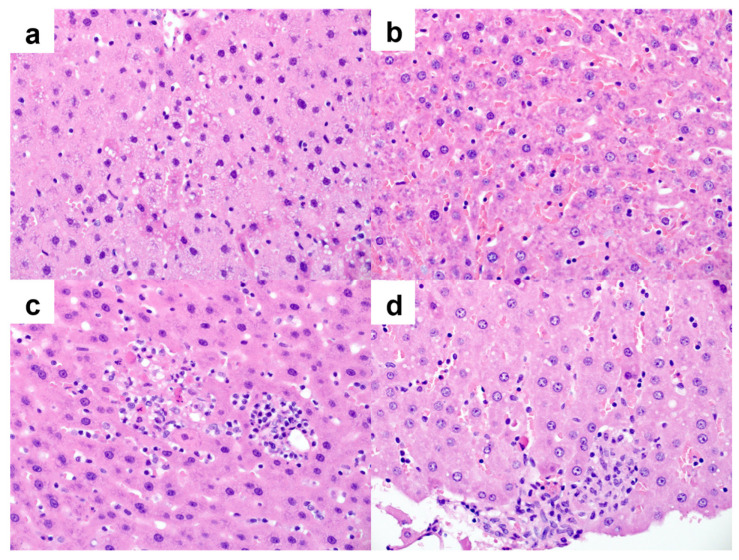
Photomicrographs taken from hepatic tissue of rats receiving GEF and ASP 175 or ASP 1000. (**a**) Mild cytoplasmic vacuolization of the hepatocytes and (**b**) sinusoidal congestion were noted in liver tissues from rats GEF and ASP 175 mg/kg and GEF and ASP 1000 mg/kg (hematoxylin and eosin, ×400 magnification). (**c**) Foci of spotty necrosis were noted in a single rat from GEF and ASP 175 mg/kg and (**d**) a non-necrotizing granuloma was noted in a rat from GEF and ASP 1000 mg/kg (hematoxylin and eosin, ×400 magnification).

Sections from liver tissues taken from rats receiving GEF and ASP 175 or ASP 1000 mg/kg showed mild cytoplasmic vacuolization and sinusoidal congestion in most rats in both groups. A single rat in GEF and ASP 175 mg/kg showed foci of spotty necrosis and another rat in GEF and Aspartame 1000 mg/kg showed non-necrotizing granulomas. Representative figures of liver histology of rat after the administration of 175 or 1000 mg/kg are shown in Figure 4.

### 2.5. Rat Body Weight

**Figure 5:**
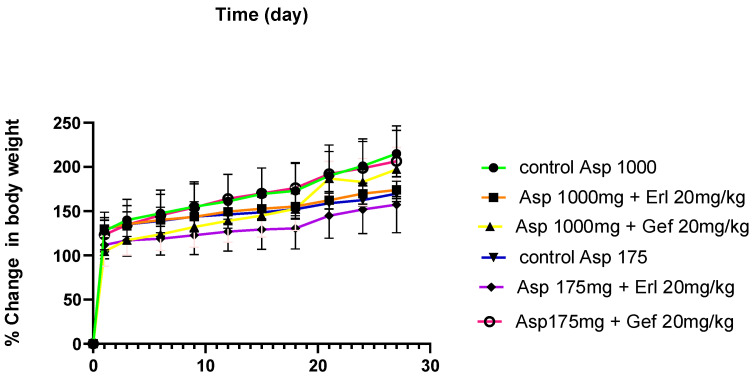
Average rat weight for all groups (*n* = 5) during the course of the study.

Figure 5 depicts the % change in body weight as the mean body weight of all groups significantly increased after for four weeks of ASP administration. There was no significant difference in the body weight among treated groups and the control. The highest recorded weight (255 g) was for the control group.

## 3. Discussion

Aspartame is an artificial sweetener used in several products, such as beverages, chewing gum, food products, and flavored water components [10]. This study was conducted as an extension to our previous work to highlight the effects of two different doses of ASP on the PKs of ERL and GEF as well as to evaluate the effect of this artificial sweetener on the liver. While both doses of ASP increased the exposure to ERL, through increasing all PK parameter of ERL except for T_1/2_ and Cl/F, ASP 1000 showed more impact in most cases. The increase in C_max_ and AUCs indicates a possible higher exposure of ERL in animals as well as a possible need to change dosing regiments. Both low and high doses of ASP decreased the C_max,_ of GEF and increased both T_max_ and T_1/2_ which indicate that it might impact the exposure of the drug as well as dosing regiments. Surprisingly, a low ASP had more impact on the apparent oral clearance and both AUCs compared to a higher dose. Since ASP is metabolized to phenylalanine and aspartic acid which bind to blood protein transporters and displace other drug, it is possible that these compounds exerted such effect on ERL and GEF altering their PKs and increasing drug exposure [6]. In addition, ASP has significantly increased on all tested liver enzymes and showed histopathological changes in liver tissues including various degrees of inflammation, cytoplasmic vacuolization, nuclear changes, and, rarely, granulomas, which indicates damage to liver tissues. It is possible that liver injury is caused by methanol, one of the byproducts of aspartame metabolism, which, in turn, is metabolized to formaldehyde and free radicals [11]. These free radicals may lead to denaturation or fragmentation of liver proteins and, thus, a possible alteration of the physicochemical properties of hepatic enzymes and loss of activities [11]. Moreover, inflamed or damaged liver cells can lead to more than usual escape of certain chemicals, including liver enzymes into the bloodstream [12]. This can, in part, explain the increase in liver enzymes and the reactive, degenerative, and inflammatory changes seen in liver tissues after ASP administration.

The impact of liver damage can also lead to alteration of drugs’ metabolism. Since the liver is the major site of drug-metabolism, any damage or alteration to liver cells or in CYP450 enzymes can lead to impaired drug biotransformation or metabolism [13]. It is well known that ERL and GEF are extensively metabolize by CYP450 enzymes, especially CYP3A4 [14]. Due to such facts, concomitant use of ERL and GEF with other drugs, dietary supplements or food that have an impact on CYP3A4 will eventually alter the concentration and exposure of these drugs [5]. Thus, increased ERL and GEF exposure in this study might be attributed to the inhibitory effect of ASP on CYP450 as our data showed.

The limitation of this study is that it reflects the scenario of the coadministration of TKIs with both low as well as high doses of ASP in rat daily after for 4 weeks of administration. The exposure due to continuous daily administration of ASP in human patient might not be as intensive as with rats. Thus, clinical evaluation of the effect of ASP on human liver enzymes and the PKs of ERL and GEF is needed.

## 4. Materials and Methods

### 4.1. Materials

ERL and GEF were obtained from Haoyuan Chemexpress Co., Ltd., Shanghai, China. Imatinib used as the internal standard (IS), Aspartame was purchased from (Sigma Aldrich, Chemie GmbH, Steinheim, Germany). Ammonium Acetate was obtained from Merck. Methanol of HPLC grade was obtained from (Panreac, Barcelona, Spain, E.U.). Liver enzyme kit (ALT, AST, and GGT) was purchased through United diagnostic industry, Dammam, Saudi Arabia.

### 4.2. Experimental Animals

Forty-five Wistar rats (male), with a weight of 250 ± 30 g were obtained from the animal house of the College of Pharmacy in King Saud University, Riyadh, Saudi Arabia. To eliminate any possible effect of the female’s estrus cycle on liver enzymes, male rats were chosen in this study [15]. Rats were randomly assigned into nine cages (*n* = 5). Cages were housed at room temperature of 25 °C and an average humidity of 50%. Observation of all rats was conducted on a daily base to ensure that all animals are in good health. All animal experiments followed the guidelines of the Ethical Committee for Performing Studies on Animals, King Saud University, Riyadh, Saudi Arabia, following protocol KSU-SE-19-13 [15].

### 4.3. Animal Study

To test the effect of ASP (low and high doses), a set of animals, 30 in total, were divided into three groups, two cages for each group (*n* = 5); group I received water, group II received 175 mg/kg of ASP, and group III received 1000 mg/kg of ASP for four weeks. One cage in each group received a single dose of 20 mg/kg of ERL and the other group received 20 mg/kg of GEF suspended in 0.5% carboxymethyl cellulose. Doses were administered to rats between 6:00 am and 7:00 am through an oral gavage. Blood samples were collected via the tail vein at predetermined intervals post drug administration as described previously. Briefly, blood samples were immediately centrifuged to collect the plasma at 3500 rpm for 15 min at 4 °C, and plasma was kept frozen at −20 °C until the time of analysis. Samples were run with a previously described method with some modifications (details can be found in Supplementary Materials). Each sample was prepared as 25 μL plasma, 30 µL IS, and the volume was made up to 100 µL by methanol. ERL, GEF, and IS concentrations were determined from rat plasma using UPLC-MS/MS system, Waters Xevo TQ-S (Waters, Singapore), equipped with Acquity UPLC C 18 column (100 × 1.0 mm^2^, 1.7 µm particle size) (Waters, Dublin, Ireland). Acetonitrile: water mixture of 80:20 (*v*/*v*) and 0.1% formic acid was utilized as a mobile phase with a flow rate of 0.2 mL/min [9]. Suitable dilution of plasma sample was made before actual analysis.

### 4.4. PK Analyses

**Figure 6:**
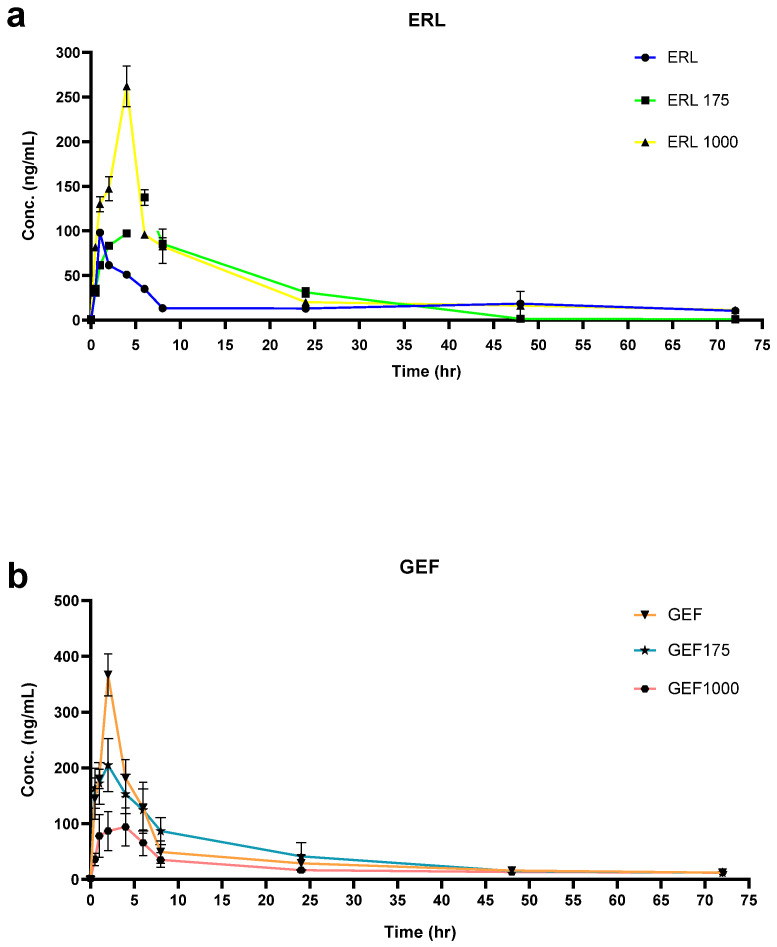
Plasma concentration–time curve after oral administration of ERL (20 mg/kg) (**a**); and GEF (20 mg/kg) (**b**) in rat receiving 175 and 1000 mg ASP (*n* = 5).

PKSolver Add-In Excel 2010, was used for PK analyses of all treated (non-compartmental analysis (NCA). Maximum concentration (C_max_) and time to maximum concentration (T_max_) were obtained from plasma concentration–time curve shown in Figure 6a,b.

### 4.5. Liver Enzyme Analysis

To evaluate the effect of ASP on rat liver enzymes, 15 rats were divided into three groups (*n* = 5), where the first group received water and served as a control, the second group received a low dose of ASP 175 mg/kg, and the third group received a high dose of ASP 1000 mg/kg once a day for 30 days [16]. Blood samples were obtained prior to ASP administration, then at one week, two weeks, and four weeks after ASP demonstration, and enzymes were measured using liver enzyme kits and following the manufacturer protocol. The effect of both doses of ASP on the level of CYP 450 enzymes using Rat CYP450 kit #. MM0247R2 (Jiangsu Baolai Biotechnology Co., Ltd., Jiangsu, China) following manufacturer protocol.

### 4.6. Liver Histology

At the end of the study, animals were sacrificed, and liver tissues were isolated and fixed in 10% formalin for 48 h. Seventy percent of ethanol was used to dehydrate the tissues, which were then sent to the Department of Pathology, College of Medicine, King Saud University for Hematoxylin and Eosin staining (H&E). Histology of liver tissues for both treated and non-treated animals were examined by two independent pathologists, through light microscopy.

### 4.7. Statistical Analysis

Results were presented as mean ± standard deviation (SD). The PKs of all treatment groups after the consumption of ASP were compared against one-way ANOVA and Bonferroni’s multiple comparisons tests using GraphPad Prism 8.0.2. Statistical significance was obtained with *p*-values ≤ 0.05.

## 5. Conclusions

The present study demonstrates the effects of administering two different doses of aspartame on the PKs of ERL and GEF and the serum liver enzymes in Wistar rate. Our results indicate that although both doses of ASP, 175 and 1000 mg/kg alter ERL and GEF PKs parameters, higher doses of ASP have the highest impact in most cases. Furthermore, a high dose of ASP (1000 mg/kg/day) can cause a significant increase in the activities of liver enzymes and reduction in CYP 450 indicating that aspartame may induce liver injury. Therefore, it might be of clinical importance to avoid the administration of aspartame containing products while patient undergo ERL or GEF therapy.

## Data Availability

Data is contained within the article and Supplementary Materials.

## Supplementary Materials

The following supporting information can be downloaded at: https://www.mdpi.com/article/10.3390/ph15111400/s1, instrumentation, chromatographic, and mass spectrometric conditions.
