# Correction: AlRasheed et al. Aspartames Alter Pharmacokinetics Parameters of Erlotinib and Gefitinib and Elevate Liver Enzymes in Wistar Rats. *Pharmaceuticals* 2022, *15*, 1400

**DOI:** 10.3390/ph18010001

**Published:** 2024-12-24

**Authors:** Hajer AlRasheed, Aliyah Almomen, Haya I. Aljohar, Maria Arafah, Rana Y. Almotawa, Manal A. Alossaimi, Nourah Z. Alzoman

**Affiliations:** 1 Department of Pharmaceutical Chemistry, College of Pharmacy, King Saud University, P.O. Box 22452, Riyadh 11495, Saudi Arabia; alrsheed891@gmail.com (H.A.); haljohar@ksu.edu.sa (H.I.A.); ranayalmutawa@gmail.com (R.Y.A.); nalzoman@ksu.edu.sa (N.Z.A.); 2 Department of Pathology, College of Medicine, King Saud University, P.O. Box 2925, Riyadh 11421, Saudi Arabia; mariaarafah@ksu.edu.sa; 3 Department of Pharmaceutical Chemistry, College of Pharmacy, Prince Sattam bin Abdelaziz University, P.O. Box 173, Alkharj 11942, Saudi Arabia; ph_m_ossimi@yahoo.com

## Error in Figure

In the original publication [1], there was a mistake in Figure 5: There was no drastic change in body weight between day 0 and day 1. Day 0 data were entered mistakenly as zero instead of the actual weight prior to administration. The text on the y axis of the graph should have indicated body weight and not % body weight. The corrected Figure 5 appears below. The authors state that the scientific conclusions are unaffected. This correction was approved by the Academic Editor. The original publication has also been updated.


