# Supplementary material for: Aspartames Alter Pharmacokinetics Parameters of Erlotinib and Gefitinib and Elevate Liver Enzymes in Wistar Rats
Source: Pharmaceuticals (Basel). 2022 Nov 14;15(11):1400. doi: 10.3390/ph15111400 (PMC9699004; doi:10.3390/ph15111400)
Supplement: Supplementary file 1 [file pharmaceuticals-15-01400-s001.zip › pharmaceuticals-1898118-supplementary.pdf]

Aspartames Alter Pharmacokinetics Parameters of Erlotinib and Gefitinib and Elevate Liver Enzymes in Wistar Rats

Instrumentation

Samples were run on UPLC–MS/MS (Waters Xevo QD separation system, Waters, Singapore,) equipped with sample and binary managers (AquityTM Ultra-performance LC) and Electrospray ionization mass spectrometry (ZsprayTM ESI-APCI-ESCI, Ultra-performance LC) with multiple reaction monitoring (MRM)-mode using a triple-quadrupole mass spectrometric detector (STEP WAVETM, Ultra-performance LC). The data were acquired and processed using the MasslynxTM Version 4.1 software (Micromass, Manchester, UK). Thirty microlites of IS working solution (50 ng/mL IS) was added to protein precipitation extract (PPE) and completed to 100 µL using methanol. Samples were then samples were then vortex-mixed for 1 min, centrifuged at 15,000 × g for 15 min, and injected into the UPLC-MS/MS system for analysis.

Chromatographic conditions

Chromatographic analysis was performed using Acquity UPLC BEHTM C 18 column (100 × 1.0 mm, i.d., 1.7 m particle size) (Waters, Dublin, Ireland). Isocratic elution was conducted with a mobile phase consisting of acetonitrile: water (80: 20, v/v), with 0.1% formic acid. The auto-sampler temperature was maintained at 10 ◦C and column temperature was kept at 45 ◦C during the runtime of 2.0 min. Samples were injected with the full loop mode at a flow rate of 0.2 mL/min

Mass spectrometric condition

Compounds were measured under positive electrospray ionization (ESI +). Acquisition was performed using multiple reaction monitoring (MRM) of the transitions from[M + H]+ to particular product ions to quantify each compound. Different MS parameters including the collision energy, capillary voltage, and cone voltage were separately optimized for each compound. The transitions used as well as the MS conditions selected for each compound were mentioned in (Table 1).

Table S1. Desolvation temperatures were separately adjusted for each compound

Target compound	Precursor ion [M + H]+	Daughter ion	Cone voltage (V)	Capillary voltage (KV)	Collision energy (eV)	Desolvation Temperature (◦C)
GEF	447.25	128.08	30	3.5	24	300
ERL	394.20	278.4	30	3.5	30	200

IMP	494.3	394.2	30	3.5	30	300

* m/z 494.3→394.2 for imatinib

A source temperature of 150 ◦C was employed. Nitrogen was used as the desolvating gas at a flow rate of 800 L/h. Collision gas flow was set at 0.15 mL/min while cone gas flow at 150 L/h. The dwell time of 0.025 s was used for all compounds. The resolutions of the MS analyzer were set as follows; LM of 2.8 and HM of 14.86 for both ion energy 1 and 2.

Calibration graphs and spiking of plasma samples

Drug free rat plasma samples spiked with predetermined amounts of each of GEF, ERL, along with IMP (IS), were used to generate matrix-based calibration data. Into a series of pre-labeled micro centrifuge tubes, portions of 0.1 mL plasma were separately spiked with different volumes of standard solutions of ERL, or GEF to get final concentrations of 0.025–100 ng/mL of. Each sample solution was also spiked with 50 L volume of 10 ng/mL IMP, being used as the IS. All samples were then made up to final volumes of 1 mL with methanol. Blank samples were prepared by adding 0.9 mL methanol to 0.1 mL plasma samples. Samples were vortex-mixed at 15000 rpm for 15 min.
